# Omnivores Going Astray: A Review and New Synthesis of Abnormal Behavior in Pigs and Laying Hens

**DOI:** 10.3389/fvets.2016.00057

**Published:** 2016-07-22

**Authors:** Emma I. Brunberg, T. Bas Rodenburg, Lotta Rydhmer, Joergen B. Kjaer, Per Jensen, Linda J. Keeling

**Affiliations:** ^1^NORSØK – Norwegian Centre for Organic Agriculture, Tingvoll, Norway; ^2^NIBIO – Norwegian Institute for Bioeconomy Research, Tingvoll, Norway; ^3^Behavioural Ecology Group, Wageningen University, Wageningen, Netherlands; ^4^Department of Animal Breeding and Genetics, Swedish University of Agricultural Sciences, Uppsala, Sweden; ^5^Federal Research Institute for Animal Health, Friedrich-Loeffler-Institut, Celle, Germany; ^6^AVIAN Behaviour Genomics and Physiology Group, IFM Biology, Linköping University, Linköping, Sweden; ^7^Department of Animal Environment and Health, Swedish University of Agricultural Sciences, Uppsala, Sweden

**Keywords:** tail biting, feather pecking, animal welfare, swine, poultry, gut–brain–microbiota axis

## Abstract

Pigs and poultry are by far the most omnivorous of the domesticated farm animals and it is in their nature to be highly explorative. In the barren production environments, this motivation to explore can be expressed as abnormal oral manipulation directed toward pen mates. Tail biting (TB) in pigs and feather pecking (FP) in laying hens are examples of unwanted behaviors that are detrimental to the welfare of the animals. The aim of this review is to draw these two seemingly similar abnormalities together in a common framework, in order to seek underlying mechanisms and principles. Both TB and FP are affected by the physical and social environment, but not all individuals in a group express these behaviors and individual genetic and neurobiological characteristics play an important role. By synthesizing what is known about environmental and individual influences, we suggest a novel possible mechanism, common for pigs and poultry, involving the brain–gut–microbiota axis.

## Introduction

Domestication, the genetic adaptation to a life under human supervision (1), has caused an unprecedented change to the life of animals. Not only have they been bred for immense changes in appearance and physiology but also the environments in which they are kept are widely remote from the natural habitats where their ancestors evolved. This has become particularly dramatic with the rise of modern agricultural practices. The housing of many farm animals today is generally characterized by relatively barren and crowded conditions, but with a high availability of standardized feed. Many animals develop abnormal behavior patterns of different kinds in these production environments.

In this review, we refer to behaviors as being abnormal if they are not normally seen in the wild ancestors in their natural habitat, and only rarely seen in domesticated conspecifics kept under less restricted conditions than those normally prevailing on farms. Abnormal behavior can actually be very common, i.e., normal in the quantitative sense of the word, among animals in specific farm conditions. In addition, our definition implies that abnormal behavior is a sign of an improper environment that the performing animal cannot cope with.

It is claimed that abnormal behavior is closely linked to a specific thwarted motivation (2). Hence, the ecological niche and natural habitats of a species can perhaps predict the kind of abnormal behavior a particular species will develop in captivity. Clubb and Mason (3) analyzed a large data set from many species of carnivores in zoos, and found that the degree of stereotyped pacing could be predicted from the typical home range size of the species in the wild. The larger the difference between their normal home range and the size of the captive enclosure, the more the animals would engage in pacing. In the same vein, it has been suggested that omnivorous and herbivorous species mainly develop oral stereotypies, such as sham chewing, bar biting, and tongue rolling, in captivity. This would reflect the thwarted motivation to explore and seek food.

In the case of pigs, there are a number of commonly observed abnormal behaviors and of those directed to pen mates, belly nosing, and biting and chewing on the ears or tail are the most commonly observed. Tail biting (TB) is a common abnormal behavior among growing pigs that involves one pig biting and chewing the tail of another, eventually causing open wounds. The behavior called “tail in mouth” is similar to TB but does not result in clinical damage to the tail. In poultry, commonly observed abnormal behaviors are feather pecking (FP), stereotyped pacing, and spot pecking. FP is a common problem among laying hens, characterized by birds pecking and pulling feathers of other hens, gradually causing large, nude skin areas and possibly bloodshed from ruptured skin follicles. In both pigs and laying hens, the injurious behaviors directed toward other individuals may escalate and develop into cannibalism, where the recipient is severely wounded or killed.

Tail biting and FP are not only detrimental for the recipients. As mentioned earlier, abnormal behavior is generally said to have its background in a behavioral need that is not fulfilled and is, therefore, a sign of reduced welfare also in the individual performing it. It is an economic problem for the farmer due to the increased use of medication, animals that die due to their injuries, as well as downgrades at slaughter. It has been reported that between 30 and 70% of European pig farms have some problems with TB (4). FP was found to have a prevalence of 86% of the flocks in UK (5).

The frequencies with which abnormal injurious behaviors are performed vary between and within groups. Some individuals become “fanatic” tail biters performing this behavior 11–25% of the time [reported in Ref. (6)]. With regard to FP, frequencies as high as 135 bouts per bird per hour (7) and three severe pecks/minute (8) have been reported. It is observed that gentle FP decreases with age, whereas the severe form increases with age and is mainly seen in adult birds (9). The same study showed that while most birds were involved in gentle FP early in life, only a small number of birds developed severe FP later in life, which was also shown by Newberry et al. (10).

Although TB and FP seem to have a great deal in common with regard to their development and possible causes and preventions, so far no one has drawn these two apparently similar abnormalities together in a common framework. The aim of this review is to do just that, in order to seek common underlying mechanisms and principles. Not only the similar appearance of the behaviors would suggest that such common mechanisms could exist, but also other peculiarities point in the same direction. For example, for both behaviors, we know that not all animals in a group are equally likely to develop injurious activity despite sharing the same environment. Also, some individuals are more likely to become victims, while others seem to be resilient to both becoming victims and performers. This raises the question whether these different categories of animals share some characteristics, and to what extent these similarities are genetic and acquired during ontogeny.

In the first part of this synthesis, we will review the present knowledge of both types of abnormal behavior and their causes and mechanisms. We attempt to cover environmental as well as genetic factors. In the second part, we suggest new research areas, in particular further investigations related to the microorganisms in the gut and its connection to development of the neurological system, that we feel might contribute to the identification of a common causal mechanism for TB in pigs and FP in poultry and which could perhaps even be generalized to abnormal injurious behavior in other omnivores.

## Pigs and Poultry – The Domesticated Omnivores

When considering possible reasons for why abnormal social behavior is common particularly among pigs and poultry, it would seem logical to consider their common ecological adaptations. Both species belong to our oldest farm animals, with a domestication history ranging about 8–9000 years back in time (11, 12). Research on free-ranging modern domestic animals shows that the fundamental aspects of their behavior are largely conserved (13, 14). This includes foraging behavior; in fact, poultry and pigs are by far the most omnivorous of the domesticated farm animal species. Both species will adapt to a wide variety of diets, including all sorts of plant-based food, insects, carcasses, and even live prey, such as small rodents.

An important aspect of successful omnivory is the need to be highly explorative. In order to survive in a habitat with constantly changing food availability, animals need to explore and exploit new food sources regularly. In semi-natural conditions, pigs spend up to half their waking time rooting and chewing in search of food (15) and zoo-kept Red Junglefowl use more than 60% of their time pecking and exploring (16). In static, barren production environments that do not allow normal exploratory behavior, pen mates are the only part of the environment that move and change.

## The Physical Environment – “Where You Are”

Traditionally, research on TB and FP has focused on how housing and management affect development of the behavior. Both TB and FP are known to be multifactorial and many risk factors have been revealed through a large number of studies aiming at prevention through keeping them in a proper environment. In this section, we will review the literature regarding how the environment may affect the development of TB and FP, although our focus is on the provision of foraging material and on nutrition.

### Environmental Conditions and Effects on Tail Biting and Feather Pecking

Modern pig and egg production are large-scale businesses performed in specialized farms and the animals are generally kept in groups.

The piglets are usually weaned from the sow at around 4 weeks of age. Rearing environment has been shown to effect TB behavior. For example, more sows per stock person, occurrence of fostering, lack of straw in the farrowing pen, slatted floor and crated sows seems to be linked to a higher risk of TB (17). A pig usually stays in a growing-finishing pen until slaughter at the age of 5–6 months. For laying hens, the chicks are transported from the hatchery to the rearing farm where they stay until about 16 weeks old. At this age, when they start to become sexually mature, they are moved to the laying hen farm where they stay until the end of their production period at 70–80 weeks of age.

In many countries, cutting the tails of all piglets is part of the management routines to prevent TB. According to the European Food Safety Authority (4), over 90% of the pigs within the European Union are tail docked, even though routine tail docking is prohibited (Council Directive 91/630/EU). Likewise, removal of the tip of the upper beak of all laying hen chicks is common practice in many countries to reduce the damage caused to other birds, although in other countries beak trimming is prohibited (18).

There are contradictory results in the literature on the effect of feed on TB. For example, survey data have shown higher levels of TB in pigs given liquid feed rather than pellets or meal (19), in pigs given pelleted rather than liquid or meal feed (17, 20), and in pigs given dry feed rather than wet feed (21). In poultry, on the other hand, the literature on FP is quite clear; fine ground (meal) feed always gives a lower risk compared to pellet feeding [reviewed by Kjaer and Bessei (22)]. For both pigs and hens, the feed is optimized for the average animal and does not take account of individual variation in nutritional needs.

One aspect of feeding that seems to be important for the risk of TB is the feeding space and competition at the feeders. The general consensus is that reduced feeding space increases the risk of TB [reviewed by EFSA (4)] possibly by causing frustration in the pigs that cannot get to the feed. These animals might then bite tails to get access to the feed (23). Interestingly, a somewhat similar effect was seen in an experiment with laying hens on the use of operant feeders (24). The hens were thought to be frustrated by experiencing (unplanned) feed restriction due to inadequate operation of the feeders and this caused FP. The effect of feeder space during the early rearing time in a pig nursery was found to have a carry-over effect on behavior during the growing–finishing period, where more TB was seen in pigs having the most restricted feeder space in the nursery (21).

Pen design and size are usually regulated by animal welfare legislation, which may differ between countries, but 0.6 m^2^ per pig is a normal figure for pigs weighing 100 kg. A high stocking density has been found to have an unfavorable effect on TB in some studies (17), but not in others (25). In many countries, the whole floor area is slatted, which hinders the use of straw (see next section). When comparing hens kept in floor pens from 15 to 120 birds, most FP activity was found in the largest groups (26).

### Foraging Material

Laying hens kept out-doors and growing pigs on pasture use a large part of the day for foraging behaviors, such as pecking and rooting (27). Already in the late 1960s, van Putten (28) suggested that since pigs are highly explorative animals, the barren production environment does not fulfill the pig’s behavioral need to root and chew, which is then redirected to the tails and ears of other pigs. This has later been supported by a large number of studies emphasizing the favorable effect of enrichment substrates, preferably straw, on TB behavior [e.g., Ref. (17, 20, 29–32)].

Likewise, one of the main hypotheses regarding the underlying biological mechanisms of FP is that it has its background in foraging motivation (33) that is not fulfilled by the environment provided. To increase time spent on feeding in the captive environment and, thus, reduce time for performing injurious behaviors, the feed can be finely ground, diluted, or supplemented with fiber and other low digestible feed stuffs [see Kjaer and Bessei (22), for a review]. The presentation of high fiber diets, such as mash and the availability of supplementary material for pecking reduced the incidence or delayed the development of FP and cannibalism in many studies [e.g., Ref. (33–37)]. It can be hypothesized that animals have a genetically determined spontaneous motivation for performing feeding-related behaviors (pecking, scratching, biting, rooting, chewing, and locomotion). This motivation might to some degree be unrelated to nutritional or other environmental factors. A mismatch between the spontaneous activity and the actual time required for feeding could be a cause for TB or FP to develop (33, 38–40).

### Nutrition

Even though foraging material is an important factor that influences abnormal behavior, it is also to be expected that there are nutritional effects. Many omnivores have the possibility to detect nutritional deficiencies and change their foraging behavior accordingly. Dietary levels of some substances can also act directly on hormone and neurotransmitter levels, so leading to increases (or decreases) in the levels of FP and TB.

#### Specific Needs and Deficiencies

The level of metabolizable energy in the diet has an indirect effect on FP [reviewed by van Krimpen et al. (41)]. It acts through the regulation of feed intake whereby a low energy diet may increase the time spent feeding as well as the physical effect of feed in the crop, gizzard, and duodenum.

It is generally acknowledged that a deficiency in crude protein (42, 43), amino acids (44, 45), and minerals (46) quickly influences the birds and elicits FP (41). Deficiencies in sodium and calcium elicit exploratory pecking (47, 48), which means birds may redirect their attention to the plumage of their pen mates and, thus, initiate FP. It is generally accepted that severe FP and cannibalism develop within a few days and affect a large number of birds when nutrient-deficient diets are fed. It usually disappears rapidly after restoring adequate nutrient levels, but feather damage developed during this period might facilitate further pecking (49).

Nutritional deficiencies in protein trigger TB in pigs in much the same way as is seen with FP in hens. A low protein diet increased TB in slatted, but not in straw-bedded accommodation (50). Since the growth rate of pigs was reduced on this diet, it seems that the protein level or quality was inadequate for optimal growth, even if the major limiting essential amino acids were supplemented to the diet. Also, giving diets more closely suited to the protein needs of the animal at any given time (phase feeding) has been shown to reduce the prevalence of TB in both survey (51) and controlled experimental comparisons (50).

Many animals, perhaps especially omnivores, will if possible increase their dietary intake of certain nutrients in the case of a deficiency and a lack of minerals, especially sodium, is said to induce TB even if most evidence of this is anecdotal (52–55). This suggests that an already existing TB problem might be alleviated by feeding supra-nutritional levels of sodium chloride. A common theme when combining pig and poultry literature is that a lack of salt increases exploratory behavior and this in turn, by chance, increases TB or FP, even if this specific behavior was not directly triggered.

#### Dietary Fiber and Gut Flora

There are changes in the intestinal tract of animals over time. For example, high relative weights of the gizzard and gizzard contents of hens have been reported after feeding various silages or carrots (37) or after supplementing wheat-based feed with coarse wood shavings (56). Interestingly, this spontaneous ingestion of wood shavings was not seen in treatments consisting of oat-based diets. In further experiments, these authors also found that ingestion of paper and feathers was higher in birds fed on low compared to high fiber diets (oat-based). So it seems that in the absence of fiber, birds eat feathers. This indicates that birds may eat feathers to compensate for the lack of structural components in the feed.

Feather eating was also studied in the FP selection lines (further on referred to as HFP and LFP lines, selected for more, respectively, less FP) developed by Kjaer et al. (57). It was found that birds from the HFP line show a special preference for feathers (58) and adding feathers to the diet seemed to reduce FP (59).

The effect of high fiber diets on FP may not only act through increasing the time spent feeding. van Krimpen et al. (60) confirmed the higher retention time of the digesta in the foregut of hens fed high fiber diets, which may give a greater feeling of satiety and may, hence, reduce FP.

In sows, dietary fiber reduces overall physical activity and stereotypic behaviors, such as bar biting and sham chewing, shortly after feeding [reviewed by de Leeuw et al. (61)]. Dietary fiber has been linked to TB but, contrary to the experimental evidence of the positive effect of fiber on FP, no experimental evidence has been published showing this effect on TB (4). It has been postulated that both low and high levels of fiber might increase the risk for and severity of TB (54). On the other hand, there is a wealth of evidence that provision of straw reduces the risk of TB (30). Even if straw has many attributes that may contribute to this reduced risk of TB, some of this straw is probably eaten; hence, providing additional fiber to the diet.

Meyer et al. (62, 63) found that the eating of feathers led to a change in intestinal microbiota, resulting in increased concentrations of keratin hydrolyzing species. This opens new avenues of research into implications of gut bacteria, their metabolites, and the polyamine system on brain and behavior in laying hens. Naturally, no such investigations exist for TB in pigs, but another mechanism could play a role for pigs, namely increased saliva production. It has been suggested that horses may show certain stereotypic behaviors, such as crib biting or wind sucking, to lower unpleasant feelings caused by peptic ulcers (heartburn) (64), the mechanism being a higher production of saliva during stereotyping that increases pH in the stomach. This is an interesting discussion, and although speculative, it may be applied also on TB pigs and FP hens. It is well known that a high proportion of pigs have ulcers (65) and it could be discussed if chewing tails increases saliva production and so stomach pH. In summary, there are indications of a role of the gastrointestinal tract in both FP and TB; however, somewhat stronger in FP. This is referred to again in Section “The Brain–Gut–Microbiota Axis: A Connecting Piece?,” which addresses the brain–gut–microbiota axis.

#### Nutritive Influences on Hormones and Neurotransmitters

Tryptophan is a precursor of serotonin, which acts as a neurotransmitter. The dietary level of tryptophan influences the level of serotonin in the brain (66) which in turn, for instance, modulates self-pecking in parrots (67, 68). Some studies have also linked serotonin to FP (69, 70) and TB (71, 72). Less feather damage was observed in bantam hens fed tryptophan levels considerably higher than recommended (22.6 versus 2.6 g/kg feed) (73). In pigs, high tryptophan levels in the feed reduced general activity (74) supporting the earlier work on the effects of tryptophan level in the feed on activity (75). In a model system, using a blood stained cloth as a model of a bloody tail (probably investigating interest in blood, rather than ongoing or future TB behavior), reduced dietary tryptophan was shown to increase this attraction to blood and to increase exploratory behavior under home pen conditions (76). Serotonin cannot pass the blood–brain barrier, but some of the tryptophan can be taken up from the gut. In both laying hens (77) and pigs (71), correlations have been found between central and peripheral measures of serotonin and these systems may influence each other through the gut–brain axis. In summary, although there are many factors in the physical environment that can influence TB and FP, numerous studies highlight the effects of the form of delivery and the nutritional content of the feed.

## The Social Environment – “Who is with You”

In the previous sections, we have discussed the effects of the physical environment on the development of TB and FP. The performance of the individual is, however, also dependent on the social environment. That is to say, changes in behavior of one individual (either in the form of the behavior or its frequency), are likely to affect the others in the group.

A pig with a bitten or bleeding tail or a wounded hen may attract interest and receive further bites or pecks as a result of its initial wounds. In poultry, it has been shown that artificially damaging feathers by cutting them attracts FP (49). In pigs, Fraser (52, 53) could show that a blood covered tail model received much more bites compared to the same tail model without blood. However, it also seems like some individuals are more likely than others to becoming a victim of TB or FP. The variation in feather condition within a group of individuals (78) shows that some individuals are receiving more injurious pecks than others. Moreover, it has been shown that a genetic mutation increases the likelihood of becoming a victim. Birds suffer more drastic FP when the color of their plumage is due to the expression of a wild recessive allele at PMEL, a gene that controls plumage melanization, and when these birds are relatively common in a flock (79). Quantitative genetic studies of FP traits show, however, that the heritability estimates of receiving FP (i.e., becoming a victim of FP) are very low, in most studies not different from zero (9, 57, 80). Also, the activity level of the victim may play a role, as passive birds are more likely to be targeted than active birds (81). In pigs, individuals could be classified as high or low receivers depending on the number of bites received (82). There is also a large variation in the response of the victim when bitten; some pigs show almost no response and others an immediate avoidance, sometimes even with vocalizations (82, 83). Furthermore, it seems that individuals that receive a high number of tail bites also receive a high frequency of other abnormal behaviors (82), although, sometimes almost all individuals in a pen/cage are receivers (8, 84).

There is no consensus on whether or not injurious behavior is socially learnt or triggered by stimulus enhancement (85). The mechanism leading to the first individuals developing this injurious behavior could be different from the mechanism for developing it later in the progress of the outbreak.

Daigle et al. (86) followed hens from 21 to 37 weeks. They found that although half of the hens were inconsistent in their behavior over time (sometimes feather peckers, sometimes victims), half of the hens were never observed to perform any FP. There seems to be a category of individuals that are in some way “resistant” to becoming directly involved in an outbreak, the so-called “neutral” animals (86, 87). Brunberg et al. (87) found that “neutral” pigs, housed in a pen with an ongoing TB outbreak, were different from pigs in a control pen without TB, despite that neither category of pig participated in actual TB behavior. They compared the behavior of neutral and control pigs and found that pigs housed in control pens performed a wider variety of pig-directed abnormal behaviors (belly nosing, tail in mouth, and “other” abnormal behaviors) compared to the neutral pigs in pens with TB. Using gene expression studies, 107 transcripts were identified as differently expressed between these two categories of pigs. Several of these transcripts had already been shown to be differently expressed in the neutral pigs when they were compared to performers and receivers of TB in the same pen (88). Hence, the different expression of these genes cannot be a consequence of the neutral pigs not being involved in TB behavior, but rather linked to the cause contributing to why they were not involved in TB interactions. These neutral pigs seem to have had a genetic and behavioral profile that somehow contributed to them being resistant to performing or receiving pig-directed abnormal behavior, such as TB, even when housed in an environment that elicited that behavior in other pigs. In laying hens, Daigle et al. (86) found that 4% of the hens were consistently neutral over time. Kops et al. (89) studied differences between feather peckers, victims, and neutral animals from the same group. They found that neutral animals differed significantly from both feather peckers and victims in brain monoamines, indicating that neutral animals indeed may form a distinct group of individuals. It seems likely that the proportion of neutral animals (pigs as well as hens) influences the social environment of all group members.

## The Individual – “Who Are You”

Different individuals seem to have different predispositions to turn to abnormal behavior to cope with their situation. Therefore, there is a large body of research regarding TB and FP that has focused on just this. The following sections will focus on reviewing the studies concerning internal factors with associations to abnormal behavior. For simplicity, these are grouped in studies dealing with sex differences, other genetic effects, personality, and the immune system. Gut microbes, and their subsequent influence on the brain (90), can also be regarded as a part of “who you are,” but because of their importance to our new synthesis of abnormal behavior we have given this gut–brain axis its own section later in this review.

### Sex Differences

Several studies in different species suggest that there is a sex influence with regard to which individuals are the most common performers or receivers of animal directed abnormal behaviors and there is a strong tendency that females are more active performers. Regarding FP, logically, most studies are performed on only females since these are the ones that are used in egg production. Jensen et al. (91) used a cross between Red Jungle Fowl and White Leghorn and could show that females were much more likely to be performers of FP compared to males. The same was observed in an experimental New Hampshire layer strain where the level of FP was low in the adult hens but practically absent in the adult roosters^1^. The studies on TB in pigs are less consistent and have usually focused on the sex of the receiver. Some studies have shown that males receive more TB than females (92, 93). The study performed by Keeling et al. (92) was performed in Swedish slaughterhouses and is, hence, assumed to comprise only castrated males, while the Zonderland et al. (93) study used boars in experimental conditions. Others did not see this difference between boars, castrated males, and females (82, 94). Yet another study suggests that (uncastrated) males are more likely to be fanatical tail biters (95).

Also females of other species have been suggested to perform more abnormal behavior. Female mice have been suggested to be approximately one and a half time more likely to perform abnormal barbering than males (96). The mechanisms behind the tendency that females of several omnivorous species seem to be more active in performing animal directed abnormal behaviors are not fully understood. However, since many of these behaviors seem to appear (or increase) around sexual maturity (48), the gonadal hormones are implicated in the behavior. Concerning FP, Hughes (97) administered gonadal hormones to pullets from the age of 12 weeks. Up to 18 weeks, progesterone produced a moderate but significant increase in pecking, estrogen, and progesterone together a much greater increase. From 18 to 24 weeks, the expected onset-of-lay rise in pecking in the controls was prevented by testosterone. There is evidence from commercial flocks that severe FP develops from around 20 weeks of age in females but not in males, further supporting the hypothesis that gonadal hormones play a role, inducing FP in females and reducing it in males.

There is also a possibility that the nutritional needs for females change when they reach sexual maturity and, hence, become more motivated to explore their surroundings to find the proper feed. Taylor et al. (6) suggested that since males and females have different dietary needs, some pigs within the group will have dietary imbalances and may, therefore, be more likely to perform TB.

### Genetics of Tail Biting and Feather Pecking

Even though pigs and chickens are generalists when it comes to foraging, more than 50 years of intensive selection for high production have turned them into specialists in meat and egg production. There is a considerable amount of research on the genetics of TB and FP. Nevertheless, so far, neither TB nor FP have played major roles in genetic evaluation and selection. Instead, the industry has tried to handle these harmful behaviors mainly by tail docking and beak trimming.

#### Correlation between Production Traits and Abnormal Behavior

It has been suggested that both TB and FP are correlated to production traits. Moinard et al. (17) found in an epidemiological study that when back fat thickness increased with 1 mm, the risk of TB decreased 1.5-fold. Breuer et al. (98) found a positive genetic correlation between TB and lean tissue growth rate and a negative genetic correlation between TB and back fat thickness. Brunberg et al. (88) also found support for a genetic association between TB and fatness. Performers and victims of TB were shown to have a different expression of the gene *PDK4* compared to neutral pigs (88). This gene is known to have impact on the fat content in pigs (99).

Contradictory to pigs, Jensen et al. (91) found that male feather peckers had a higher body fat percentage compared to male non-feather peckers. However, the most important selection traits in laying hens are those related to egg production and, in the same study, they found that female feather peckers started laying eggs earlier compared to non-feather peckers. Su et al. (100) found that birds from a LFP line had a lower growth rate, a larger total egg mass and a lower residual feed consumption (more efficient) compared to a HFP line. The better feed efficiency in the LFP line was related to a lower energy requirement for maintenance and this was only partly explained by a better plumage cover. Bennewitz et al. (80) found that the genetic correlation between FP and egg production was 0.50. Thus, for both pigs and laying hens, there is evidence that the selection for high production has led to changes in behavioral traits with effects on TB and FP.

#### Breed Differences and Heritability

A first indication of a behavior being determined by genetics is differences between breeds or lines and several studies have shown that different hybrids of hens show different amounts of FP (45, 101–103). There are also indications that TB is more common in certain breeds (94, 104, 105).

Heritability studies in both hens and pigs have contributed to knowledge regarding the genetic influences on these traits. The heritability estimates for FP vary between 0 to 0.15 (106), 0.05 to 0.38 (107), 0.11 (80), and 0.56 (108) and for TB between 0 in Large White pigs and 0.27 in Landrace pigs (98). That the heritability for FP is moderate has been further demonstrated in the selection lines created by Kjaer et al. (57), in which a significant difference in the level of FP could be seen between the HFP and LFP lines after only two generations. Realized heritability was estimated to 0.20.

#### Molecular Genetics of FP and TB

The first studies on molecular genetics underlying TB and FP were QTL studies of FP. In the first studies by Buitenhuis et al. (109, 110), a cross between two White Leghorn lines differing in FP behavior was used. A QTL analysis of the F2 generation resulted in a few significant/suggestive QTLs for performing severe/gentle FP. Jensen et al. (91) used a cross between Red Junglefowl and White Leghorn and found one suggestive QTL for performing severe FP that was not overlapping with the ones found by Buitenhuis.

Biscarini et al. (111) used a single-nucleotide polymorphism (SNP) assay to perform an across-line association study of FP. The study identified 57 SNP markers with an associative effect of cage mates on the individual’s plumage condition, which would reflect the genes predisposing an individual to perform FP. The authors suggested an involvement of the serotonergic and immune systems based on the functions of some of the genes. Interestingly, the lines used were also found to differ both in feather damage (103) and in serotonergic parameters (77). Flisikowski et al. (112) performed a candidate gene approach and presented molecular genetic evidence that the serotonergic and dopaminergic systems may be involved in FP.

A genome-wide association study using SNP markers was performed for TB in pigs (113), in which one significant association with being the performer of TB was found.

The predisposition to be victims of TB and FP also has a genetic background. Some QTLs have been associated with being a victim of FP (79, 109). The QTL identified by Keeling et al. (79) included the causative gene *PMEL*, encoding a protein known to control plumage color. It was shown that pigmented birds were more vulnerable to receive pecks. In the study by Biscarini et al. (111), 11 SNPs with direct effects on own feather condition was suggested to reflect susceptibility to being a victim of FP. In the genome-wide association study in pigs by Wilson et al. (113), several significant associations with being a victim of TB were found. However, the trait “being a victim of injurious behavior” might be dynamic and highly influenced by the actual environment. For example, a certain feather color pattern will attract more attention and, thus, induce more pecking when its frequency in the population is low (it stands out) compared to when it is high.

A few expression studies have been performed in FP hens. Brain gene expression differences in high and moderate feather peckers were investigated by Labouriau et al. (7) and 456 genes were differently expressed between the two bird categories. Hughes and Buitenhuis (114) used the same HFP line as Labouriau et al. (7) and compared this with the LFP and their control line, all three lines originating from the same population (57). However, they found no differently expressed genes between lines. Instead, they focused on the reduced variance in gene expression in the HFP line and found that several genes with roles in nervous system development and immune mechanisms were associated with the level of FP. Brunberg et al. (8) used birds from a commercial farm (LSL strain). Sixteen genes were differently expressed between peckers, victims and controls. The genes had functions in immune mechanisms, glucose metabolism, and intestinal bowel disease. Wysocki et al. (115) investigated gene expression variability between high and low FP groups and found 313 signals significant for a fold change higher than two. A subset of functional candidate genes confirmed these changes for four genes (HTR1B, SIP1, PSEN1, and GLUL) important in neurotransmission and psychopathological disorders.

Similar studies were done on TB in pigs. Brunberg et al. (87, 88) reported results from two studies, although including the same animals, in which brain gene expression in matched quartets of tail biters, victims, neutral pigs from the same pen, and control pigs from a pen without TB were compared. The most important conclusion from these two studies was that the neutral pigs differed most in both gene expression and behavior from all other categories. Among the genes that were differently expressed in the neutral pigs compared to all other categories were genes with a known influence on fat content, social behavior, and novelty seeking. Results from Kops et al. (89) on brain monoamines in laying hens suggest that also in hens the group of neutral individuals may indeed be a very interesting and relevant population to investigate further. Understanding why specific individuals do not get involved in TB or FP may be a key to solving the problems.

### Personality and Coping

It has been suggested that individuals with a certain personality or coping strategy in stressful situations are more likely to develop abnormal behavior (116). A coping strategy predisposes how an individual responds to environmental challenges and consists of behavioral, physiological, and neurobiological characteristics (117). Animals with a proactive strategy are more likely to develop behavioral pathologies and, therefore, it has been suggested that feather peckers have a proactive coping strategy. Some studies based on two lines selected on production parameters but by chance differing in the level of FP support this. It was shown that high pecking hens showed several of the characteristics known to be associated with a proactive coping strategy, such as lower levels of corticosterone (70, 118), higher plasma nor-adrenalin levels (118), and lower heart rate variability (119, 120). But the findings could only in part be supported by experiments using hens from lines selected specifically for more (HFP), respectively, less (LFP) FP. In another study, contrary to that expected from the coping hypothesis, higher corticosterone levels after physical restraint were found in HFP compared to LFP line birds (121). Similarly, selection on low mortality in group-housed laying hens resulted in a higher corticosterone response in the control line than in the low mortality line, which also showed less injurious behavior (122, 123). Thus, although there seems to be links between coping strategy and the development of injurious social behavior in poultry, their interpretation is far from clear.

Also in pigs, active and passive coping styles have been proposed, based on piglets’ reaction in a back-test (124). Back-test response has a high heritability and is genetically correlated to growth rate (125). Pigs with a low resisting response in the back-test spent more time manipulating pen mates (126) but no genetic relationship between the back-test response and TB has been reported.

Fearfulness can be defined as the propensity to be more or less easily frightened (127). In laying hens, varying associations have been found between fearfulness and FP. Evidence has been found that fearfulness may predispose animals to become feather peckers (9, 128, 129). Jones et al. (128) investigated fearfulness in two lines showing differences in FP behavior and found that birds from a low FP line were more active in an open-field test compared with birds from a high FP line. It was suggested that this might be an indication of reduced fearfulness in the low FP line. Rodenburg et al. (9) studied the relationship between open-field response and FP at an individual level and found a strong genetic correlation of −0.65 between open-field activity at young age and pecking behavior at adult age. A laying hen line selected for low mortality due to cannibalism had reduced fear levels in an open-field test at 5 weeks of age, compared to a control line (123). These differences were observed before severe FP and cannibalism developed, so they cannot be the result of differences in levels of pecking behavior between lines. Grams et al. (130) found a positive genetic correlation (0.20) between tonic immobility (TI) duration in juvenile hens with FP behavior in adult hens. However, behavioral tests performed on the HFP and LFP lines, i.e., the lines originally selected on FP, showed no differences in fearfulness (131) or indicated lower fear in the HFP line (132).

### Immune System

Relationships have been found between injurious behavior and the immune system. In this complex relationship, also the ability of an individual to cope with fear and stress may play a role, as it is known that stress can suppress the immune system (133). In laying hens, Buitenhuis et al. (134) found a strong genetic correlation between severe FP and immune response to keyhole limpet hemocyanin antigen. Furthermore, it has been found that immunization with human serum albumin (HuSA) at young age, a procedure quite similar to a routine vaccination, predisposes birds to develop FP as adults (135). Interestingly, selecting on low mortality due to cannibalism leads to a less pronounced immune response to a HuSA challenge in adult birds (136). Similarly, line differences in immune response were found between the HFP and LFP lines, with the LFP line having a better immune competence (number of white blood cells and the expression of MHC class I molecules on CD4, CD8β and on B cells) (137). Furthermore, Brunberg et al. (8) found changes in gene expression of genes related to immune responses when they compared feather peckers, victims, and control birds, as did Biscarini et al. (111) in a genome-wide association study.

Pigs selected for a positive effect on the growth of their group mates were found to have a lower leukocyte, lymphocyte, and haptoglobin concentration than pigs with a negative effect, indicating that also here stress and immune indicators could play an important role (138). Camerlink et al. (139) found that selection for a favorable genetic effect on growth of group mates reduced biting behaviors. Moinard et al. (17) found a correlation between disease prevalence and TB at a farm level, concluding that there may be an association between healthy animals and low incidence of TB. Scollo et al. (140) reported that pigs with access to straw showed less TB and lower blood haptoglobin, compared to pigs without access to straw. A better understanding of the relationships between TB or FP, stress, and the immune system could be of vital importance for understanding these problems.

## The Outcome – Injurious Behavior Develops in the Group

In this part of the paper, we discuss ontogeny of the abnormal behavior, from the first subtle changes in the behavior of an individual to the full outbreak of TB or FP. There have been studies where it has been possible to look back through data collected before an outbreak of injurious behavior that resulted in damage to other individuals in the group, to follow the (unsuccessful) attempts by the animals to cope with group life. Most of these data are on production-related traits recorded at testing stations used in breeding programs and so far, this has only been done in pigs [e.g., Ref. (94, 105)]. For the main part, these studies support the work of Van de Weerd et al. (95) finding that future tail biters are or tend to be lighter, and that future victims are or tend to be heavier than other individuals in the group. Average daily weight gain, however, is lower in victims once they have been tail bitten (141). Using data from automatic feeders, Wallenbeck and Keeling (142) were able to show that low feeding frequencies observed at pig group level may predict pens that will have tail damage due to TB already 9 weeks before the actual tail damage is observed. At the individual level, within a future TB pen, the number of visits to the feeder per day was greater for future TB victims 2–5 weeks before the start of the TB. Zonderland et al. (143) observed pens 6 days before the outbreak and found that future biters tended to spend more time sitting and kneeling, whereas future victims more frequently changed position. Changes in tail posture, so that the tail is hanging instead of being curled, may reflect the initial stages of tail damage, but they can also predict tail damage 2–3 days later (144).

The systems used for egg production make it difficult to follow the individual bird in the same detail as with pigs. But in an experimental setup, with rearing in large floor pens, Newberry et al. (10) found that more “foraging” and “walking” in young females were significant factors predicting FP as adult hens. This closely resembles locomotor activity recorded in the study by Kjaer (145), where it was found that the HFP and LFP lines differed in locomotor activity in their home pen, with HFP hens being more active.

These results suggest that even if there are factors predisposing animals to become performers or victims, there are changes in behavior in the days and weeks before the outbreak that are unique to those particular individuals starting to develop abnormal behavior. These changes could be used not only to predict the outbreak but also to predict how the individual will be affected by the outbreak.

## The Brain–Gut–Microbiota Axis: A Connecting Piece?

As seen in the previous sections, decades of research on TB and FP have resulted in a large amount of information about the possible causes. Several factors are, hence, known to influence the occurrence of these behaviors, including environmental (such as feed), individual (i.e., genetics, neurobiology), and social factors (group dynamics). Despite this knowledge, the link between these factors seems to be missing. Hence, understanding on how these factors are connected is insufficient. Within human psychology, there is an ongoing discussion on the relationship between stress responses and gut microbiota, called the brain–gut–microbiota axis, see, for example, Dinan and Cryan (90). They indicate a function of gut microbiota in early programing of HPA-axis activity, as it is known that gut pathogens, such as *Escherichia coli*, can activate the HPA-axis (146). On the other hand, presence of some types of gut flora, such as Bifidobacterium infantis, can have positive effects, similar to treatment with probiotics. In rodents, an increase in this specific strain of Bifidobacterium also resulted in an increase in tryptophan, a precursor of serotonin (a central neurotransmitter in the gut–brain axis) (147). Treating rats with probiotics after maternal separation normalize basal cortisol levels, which otherwise are elevated (148). Activation of the HPA-axis increases gut permeability and can result in activation of the immune system (149). Data from human studies confirm these relationships, linking depression to irritable bowel syndrome, enhanced gut permeability and increased HPA-axis reactivity. In both rodents and humans, there are ongoing studies focusing on the potential for treating subjects/depressed patients with probiotics [reviewed by Foster and McVey Neufeld (148)].

Desbonnet et al. (150) showed that gut microbiota composition influences social and repetitive behavior in mice. Germ-free mice, e.g., without gut microbiota, show social impairments in behavioral tests. Moreover, they also spend an increased amount of time engaged in repetitive self-grooming behavior during a social interaction test. However, following post-weaning bacterial colonization of the gut of the germ-free mice, these behaviors were normalized. The authors concluded that microbiota are crucial for normal social behavior and are important regulators of repetitive behavior.

The relationships between gut microbiota, the immune system, HPA-axis reactivity, the ability to cope with challenges, and behavior may play a key role in omnivorous species developing injurious behavior. Being a successful omnivore means that the feed intake, and probably also the gut microbiota, is much more varied than in herbivores or carnivores. In the commercial environment, pigs and layers are prevented from ingesting the large variety of feed that they would have done in the wild. Thereby their natural variety in gut microbiota may also be reduced and the balance between different types of bacteria may have shifted. The microbial diversity may change during life, but it is to a large extent established during early life. Studies of mice (151, 152) and humans (153) suggest that the genotype of the host influences the establishment of the microbiota. Benson et al. (151) studied mice and identified 18 host QTL associated with the abundance of various species in the microbiota.

For piglets to survive and to develop both gut microbiota and an effective immune system, it is absolutely essential that they ingest enough colostrum during their first hours. It has been shown that individuals receiving less than 290 g colostrum have a 15% reduction in post-weaning body weight (154). Moreover, Di Giancamillo et al. (155) showed that probiotic ingestion before weaning had a positive effect on post-weaning weight gain and intestinal immune function. Hence, it is tempting to speculate that the colostrum intake influences the gut–brain–microbiota axis and also has an effect on stress susceptibility and TB, but this needs further investigation. The same may apply to early intake of concentrates. van Nieuwamerongen et al. (156) recently showed that rearing piglets in a multi-litter system lead to earlier intake of concentrates by the piglets, less diarrhea, and reduced manipulative behavior.

In commercial laying hen practice, outbreaks of severe FP are frequently reported to coincide with or follow *E. coli* infections or cases of chronic enteritis. Also as reported earlier, relationships are reported between FP and the immune system. Parmentier et al. (135) found that triggering specific immunity in young birds, similar to standard vaccination programs, resulted in them being more likely to develop severe feather damage later in life compared with untreated controls. Regarding TB, we are not aware of any studies investigating the association to bacterial infections, although there is evidence that certain health problems are more common on farms with TB problems (17). Also, Reimert et al. (138) found that pigs selected for better group performance showed less TB and also differences in stress- and immune-related traits compared with pigs selected for poorer group performance. Links between performance of TB or FP and the immune system in gene expression studies have already been reviewed in a previous section.

As earlier described, there seems to be an effect of sex on the risk of developing TB and FP. It has earlier been suggested that one reason for this could be that males and females have different dietary needs, which may increase the likelihood for some individuals to develop abnormal behavior (6). Bolnick et al. (157) could show that there is a G × E interaction influencing the effect of a specific diet on gut microbiota in both fish and humans. The authors state that this may not only have an impact of the design of future studies regarding how diet affects gut microbiota, but also that it might explain the sex-biased rate of diseases that may be affected by microbiota. If TB and FP is influenced by the brain–gut–microbiota axis, it should be investigated if this diet × gender interaction can explain the differences in TB and FP between males and females.

Christian et al. (158) could show that differences in microbiota in young children were associated with different temperament traits, such as extraversion, effortful control, and fear. The differences in microbiota could not be explained by differences in diet. That is, it seems like differences in personalities and in the microbiome might be correlated, which suggests yet another link between TB and FP, since these both seem to be linked to personality. Christian et al. (158) saw some of the correlations between temperamental traits and gut microbiome composition only in males and others only in females, supporting the observation of a G × E interaction of Bolnick et al. (157).

In the gene expression study by Brunberg et al. (8), two genes known to be involved in inflammatory bowel disease (IBD) were differently regulated between feather peckers, victims, and control birds. *ABCB1* showed to be upregulated in peckers compared to both victims and control and TNSF15 in peckers compared to victims. The authors highlighted the link between IBD and osteoporosis/weak bones, which in turn has been linked to FP (91). Noteworthy also is that it has been suggested that IBD might be a result of a dysfynction of brain–gut interactions and that the disease has a psychoneurological basis (159, 160).

Meyer et al. (63) showed that HFP birds seem to have a different gut microbiota than LFP birds. They also found that feather eating affected the composition of the gut microbiota (62). Furthermore, HFP birds were more likely to eat feathers than LFP birds (161). Ingestion of feathers had positive effects on feed passage rate, similar to the effect of feeding insoluble fiber (162). Furthermore, ingestion of feathers and/or fiber (for instance, through eating straw in pigs) will result in increased gut wall stimulation, resulting in an enhanced release of serotonin from the gut wall and altered serotonin signaling throughout the gut–brain axis (163). As mentioned in Section “Nutritive Influences on Hormones and Neurotransmitters,” both FP and TB seems to be associated with serotonin.

Given the above discussion, the specific hypothesis to be tested is whether pigs that show TB have a different composition of gut microbes than pigs that do not develop the behavior. If this is found, then the next step, given the bidirectional nature of the gut–brain axis, would be to determine if the microbiota were different already early in life, i.e., even before the pig developed TB, thus paving the way for a potential link to early colostrum or feed intake or rooting behavior on how well the individual later copes with its situation. In birds, as mentioned above, it has already been shown that HFP hens seem to have a different gut microbiota compared to LFP hens and that feather eating changes the microbiota (62, 63). It should be investigated if FP individuals from commercial stocks differ from non-FP birds in the same flock. Also, it should be investigated whether this difference is apparent even before the bird starts to show FP, i.e., starts feather eating. If differences in the microbiota are found, the next step may be to try to manipulate the gut microbiota, through, for example, probiotics, in both pigs and poultry. Another future possibility may be to select pigs and poultry with favorable microbiota.

The above evidence supports an involvement of the brain–gut–microbiota axis on the development of FP and TB. Genetic make-up and early feed/microbiota intake, as well as present environment may have an effect on how the individual copes with environmental and social challenges. These challenges may in turn have an effect on the gut microbiota and stress coping. We have proposed some specific hypotheses to be tested, the first being to investigate at the individual level, whether pigs and poultry showing TB and FP do have a different composition of gut microbes compared to other individuals then, second, if these differences exist already in the young piglet or chick. Such a finding would support the mechanism proposed by Dinan and Cryan (90) whereby gut microbiota act on the adrenal cortex and then via neurotransmitters, including serotonin, to the brain so predisposing the individual to develop abnormal behavior. Although whether TB or FP actually develop will depend on the environment. If no differences in gut microbes are found before the behavior develops, then this may implicate the reverse mechanism, also proposed by Dinan and Cryan (90), whereby it is the stress of the outbreak that promotes an increasing gut “leakiness” leading to an increase in pro-inflammatory cytokines. In this case, it would be expected that all individuals involved in the outbreak, and not only the performers, will have a gut microflora composition that deviates from neutral individuals. Whatever the main direction of the mechanism in the brain–gut–microbiota axis for the different categories of individuals, we would expect similarities between pigs and poultry.

## Synthesis

**Figure 1:**
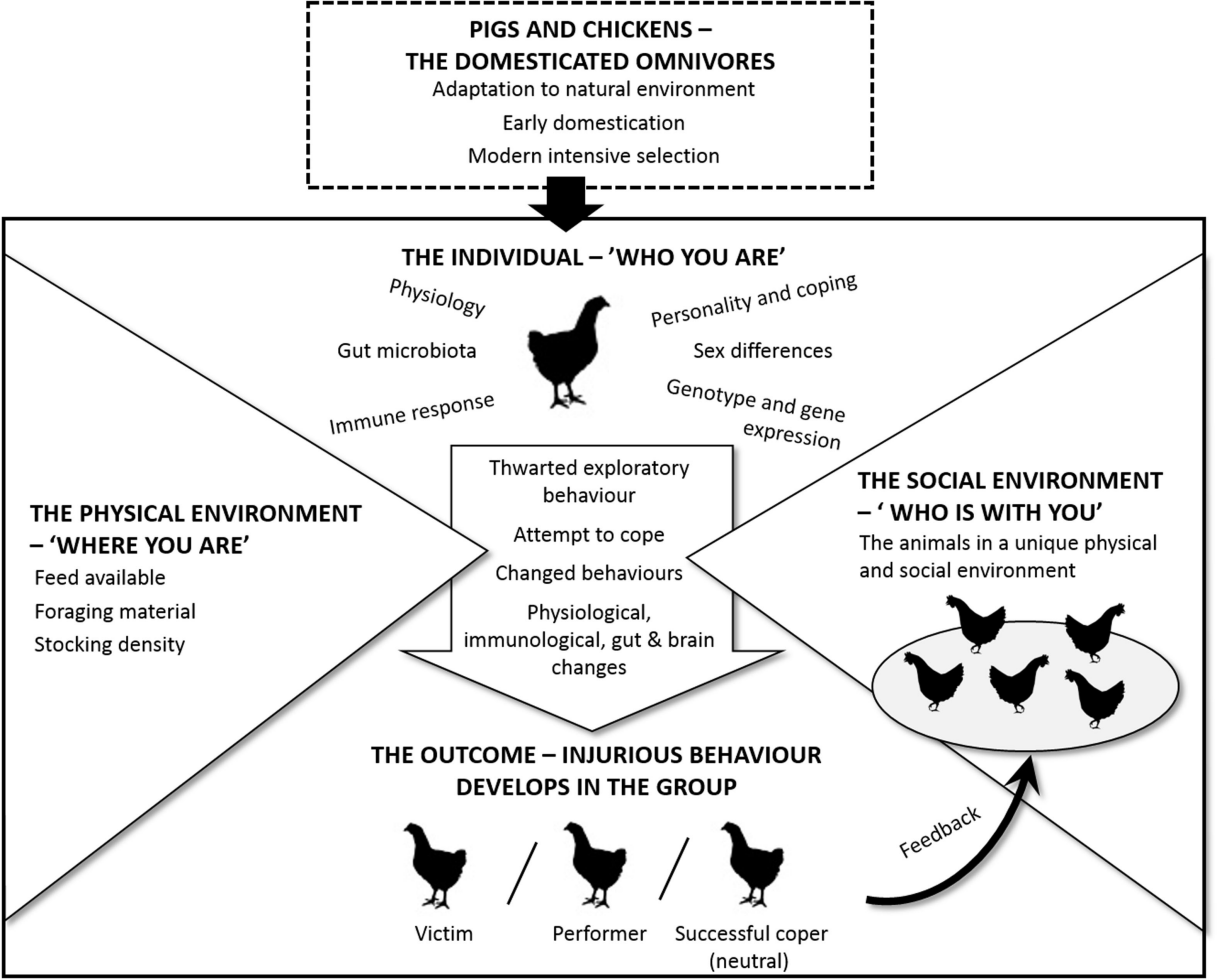
**Schematic overview on how the development of TB in pigs and FP in hens are affected by species and individual characteristics as well as the physical and social environment**.

Previously, in this paper, we have presented evidence regarding the causes of TB and FP. In addition to the ideas and hypotheses proposed by others, we have drawn on new sources of knowledge from a wide range of disciplines. For example, there have been exciting developments recently, outlined in the previous section, related to how commensal organisms in the gut play a role in early programing and later responsivity of the stress system. A novel and key aspect of our approach is to integrate information on both TB and FP in the search for similar underlying principles or mechanisms. Our intention is to contribute to a framework that can help structure future efforts to prevent these abnormal behaviors in practice. This is summarized in Figure 1.

A starting point is that both pigs and poultry are omnivores (top of Figure 1) and that despite intensive selection on production-related traits, there has been little change in their behavior. As omnivores, they are particularly adept at exploring the environment to select the most appropriate diet for their specific nutritional needs. In precocial species, this early exploration in a natural environment could be expected to play an important role in the development of a normal gut microbiota. A second point, indicated by the arrow in the center of the figure, is that individuals vary in “who they are” and as such respond differently to the external pressures from “where they are” (the physical environment) and “who is there with them” (the social environment). Individuals may, therefore, experience the same physical and social environment differently depending on their individual genotype, nutritional needs, early experiences, their social rank, and so on.

An individual pig or chicken will show unique behavioral, physiological, immunological, gut, and brain changes as it attempts to cope with its situation. Furthermore, although it is well established that the brain regulates gut activity, there is evidence that gut microbes influence brain function, especially areas of the brain devoted to stress regulation, affecting production of neurotransmitters and, hence, the animal’s behavior. Consequently, the animal and its microbiota (the brain–gut–microbiota axis) should be studied together when trying to learn more about FP and TB. We foresee an increasing demand for metagenomic analyses of gut flora in individual hens and pigs related to their specific behavior.

Since each group of hens or pigs consists of different individuals, the processes within a group are unique to that specific group. That is to say, changes in the behavior of one individual are likely to affect the others in the group in a dynamic fashion. As can be seen from the bottom of Figure 1, there are three potential outcomes for an individual when its specific characteristics are exposed to the pressure from the environment and the group. It can either remain neutral or become a performer or a victim. The neutral individual can be considered a successful coper. However, for at least some individuals, the outcome of the physical and social pressures is that they start to perform an injurious abnormal behavior. The exact form of this may vary, but a common characteristic for both types of damaging behavior is that they seem to be similar to foraging and feeding behavior. This, we have argued throughout this paper, may be the reason why social omnivorous species are more prone to develop these behaviors than herbivores, such as horses and cattle, and why we emphasize the potential importance of the brain–gut–microbiota axis.

In the future, metagenomic studies of microbiota may reveal genetic variance in the ability of pigs and poultry to provide a favorable environment for a microbiota composition associated with a low degree of abnormal behavior. That could pave the way for new selection traits. Attempts to solve TB and FP problems by improving the environment (be it housing, feeding, or other management routines) should be performed with the individuals, including their microbiota, in mind. We suggest that future research should focus on the links between known environmental and social factors affecting TB and FP, and the brain–gut–microbiota axis. The impact of gut microbiota on immune parameters, the HPA-axis, the serotonergic system, and the development of injurious behavior should be tested in experimental studies. Both differences in microbiota before, during, and after FP or TB outbreaks and differences between performers, victims, and neutral animals should be studied.

In summary, evidence linking the brain–gut–microbiota axis to abnormal behavior is perhaps most convincing in poultry, but there is enough supporting evidence from pigs to make this a fruitful area of research in the future.

## Author Contributions

All authors discussed the ideas presented in the review and participated in drafting, writing, and editing the manuscript.

## Conflict of Interest Statement

The authors declare that the research was conducted in the absence of any commercial or financial relationships that could be construed as a potential conflict of interest.

## Footnotes

^1^Kjaer (unpublished data).
